# Association between *KCNJ11* rs5219 variant and alcohol consumption on the effect of insulin secretion in a community-based Korean cohort: a 12-year follow-up study

**DOI:** 10.1038/s41598-021-84179-9

**Published:** 2021-02-25

**Authors:** Ji Ho Yun, Min-Gyu Yoo, Ji Young Park, Hye-Ja Lee, Sang Ick Park

**Affiliations:** Division of Endocrine and Metabolic Diseases, Center for Biomedical Science, Korea National Institute of Health, Cheongwon-gun, Chungcheongbuk-do 363-951 South Korea

**Keywords:** Genetics, Genetic association study, Genotype

## Abstract

Chronic alcohol consumption is known to be associated with type 2 diabetes (T2D), which is developed by two underlying mechanisms, β-cell dysfunction and insulin resistance. Identification of genetic variants in association with the development of T2D may help explain the genetic risk factors of T2D. In this study, we tried to find out some genetic variations, which interact with alcohol consumption and also are associated with β-cell function through 12 year’s follow-up study in Korean population. We performed a genotype association study using the community-based Ansung-Ansan Cohort data (baseline *n* = 3120; follow-up *n* = 433). Genotype association analyses of the baseline data showed that alcohol consumption is associated with the decreases of blood insulin levels and insulin secretion in participants with the *KCNJ11* rs5219 risk allele. Moreover, multivariate logistic regression analyses revealed that the risk allele group is vulnerable to impairment of β-cell function in response to alcohol consumption (OR 1.450; 95% CI 1.061–1.982). Furthermore, 12-year’ follow-up results showed that alcohol consumption synergistically decreases insulin secretion in participants with *KCNJ11* rs5219 risk alleles. Our findings demonstrate that the *KCNJ11* rs5219 risk allele in combination with alcohol consumption could be a potential risk factor of β-cell dysfunction. We hope that this new findings could be helpful to further understand the development of T2D depending on individual genetic background in association with alcohol consumption.

## Introduction

Type 2 diabetes (T2D) is a major global health problem with long-term consequences^1^ caused by insulin resistance and/or abnormalities in the production and secretion of insulin. T2D is known to occur through complex interplay including the interaction of genetic and environmental factors^2^.

One of such environmental factor is alcohol consumption, which is closely associated with the risk for T2D and other diseases^3,4^. Several studies have suggested that the risk for T2D is associated with alcohol consumption patterns; heavy alcohol consumption has been shown to increase the risk for T2D incidence. On the contrary, the effects of light and moderate alcohol intake are sometimes protective^3,5,6^. In line with theses, we have recently reported that chronic alcohol consumption potentiates the reduction of insulin secretion caused by pancreatic β-cell dysfunction^6,7^. To more clearly understand the underlying mechanism, we investigated the involvement of genetic factors.

The pancreatic beta-cell KATP channel subunits Kir6.2 (*KCNJ11*) gene encodes the inwardly rectifying K^+^ channel (Kir6.2) subunit, a component of the K_ATP_ channel together with the high-affinity sulfonylurea receptor 1 (SUR1), which plays a central role in glucose-stimulated insulin secretion from pancreatic β-cells^8^. Several studies have shown that genetic variations, including single-nucleotide polymorphisms (SNPs), are related to the development of insulin resistance and T2D^9,10^. Among the SNPs in the *KCNJ11* gene, rs5219 is associated with increased risk for T2D in various populations, although the findings in Asian populations are inconsistent^11–13^. *KCNJ11* rs5219 is a common variant in which substitution of C to T replaces glutamate with lysine at position 23 (E23K) in exon 1, causing a decrease of insulin secretion. The SNP may change the charge of the ATP-binding region and reduce channel sensitivity to ATP molecules, resulting in over-activation of the channel without affecting the structure of the protein^11,14^.

Despite abundant evidence that the *KCNJ11* rs5219 variant is directly or indirectly related to insulin secretion and the incidental risk for T2D risk, the relationship between *KCNJ11* rs5219 and alcohol consumption on insulin secretion has not been studied. Therefore, we investigated the insulin secretion in association with genetic variation in *KCNJ11* and alcohol consumption in a community-based Korean cohort through a 12-year follow-up study.

## Results

We preliminarily analyzed the association between common variants of *KCNJ11* (Supplementary Table 1) and T2D risk in community-based Ansung-Ansan Cohort data. Most common variants were associated with T2D risk but there was no association with insulin secretion (data not shown). However, when we analyzed the association with alcohol consumption, a few variants of *KCNJ11* including rs5219 were marginally associated with β-cell function of drinkers. Among them, rs5219 (β − 3.834, *p* = 0.0490), a causal variant of *KCNJ11* gene, was used in the further study.

### General characteristics of participants

The general characteristics of the participants according to *KCNJ11* rs5219 genotypes are shown in Supplementary Table 2. We found no significant differences in age, body mass index (BMI), systolic and diastolic blood pressure, fasting glucose, high density lipoprotein (HDL)-cholesterol, total cholesterol, aspartate transaminase (AST), or alanine aminotransferase (ALT) levels among *KCNJ11* genotypes; however, triglyceride levels differed among the *KCNJ11* rs5219 genotypes. The mean 60 min insulinogenic index (IGI_60_) values were not significantly different among genotypes (CC allele, 9.0 ± 9.0; CT allele, 8.7 ± 9.0; and TT allele, 8.7 ± 9.1). Table 1 shows participants’ characteristics according to alcohol consumption status. Systolic and diastolic blood pressure, fasting glucose, HDL-cholesterol, triglycerides, total cholesterol, and AST levels were significantly higher in drinkers than in abstainers (*p* < 0.05), and the mean IGI_60_ was 9.3 ± 8.7 for abstainers and 8.6 ± 8.2 for drinkers (*p* < 0.05). However, we found no associations between alcohol consumption and IGI_60_ according to *KCNJ11* rs5219 genotype (CC allele: abstainers, 9.3 ± 9.3 vs. drinkers, 8.9 ± 8.8; CT allele: abstainers, 9.4 ± 9.7 vs. drinkers, 8.4 ± 8.7; and TT allele: abstainers, 9.1 ± 10.0 vs. drinkers, 8.5 ± 8.6). The prevalence of T2D was not significantly different between abstainers (8.7%) and drinkers (8.7%).

**Table 1:** Participant baseline characteristics according to alcohol consumption status.

	Abstainers	Drinkers	*p*-value
Number of subjects (%)	915 (29.3)	2205 (70.7)	
Age (years)	51.9 ± 8.9	52.5 ± 8.9	0.0997
Body mass index	24.7 ± 3.2	24.5 ± 3.1	0.0917
Systolic blood pressure (mmHg)	120.7 ± 16.7	122.7 ± 17.2	0.0022
Diastolic blood pressure (mmHg)	80.4 ± 10.5	82.7 ± 11.1	< 0.0001
Glucose (mg/dL)	85.7 ± 14.9	89.4 ± 19.2	< 0.0001
HDL-cholesterol (mg/dL)	40.2 ± 8.6	44.9 ± 10.1	< 0.0001
Triglycerides (mg/dL)	160.0 ± 93.0	184.5 ± 126.0	< 0.0001
Total cholesterol (mg/dL)	189.5 ± 35.0	193.7 ± 36.4	0.0032
AST (IU/L)	29.8 ± 13.9	33.7 ± 21.9	< 0.0001
ALT (IU/L)	32.1 ± 25.4	33.9 ± 27.0	0.0913
IGI_60_	9.3 ± 8.7	8.6 ± 8.2	0.0460
*KCNJ11* (rs5219) CC	9.3 ± 9.3	8.9 ± 8.8	0.5209
*KCNJ11* (rs5219) CT	9.4 ± 9.7	8.4 ± 8.7	0.0741
*KCNJ11* (rs5219) TT	9.1 ± 10.0	8.5 ± 8.6	0.4697
Type 2 diabetes (%)	75 (8.7)	181 (8.7)	0.9688

Data are expressed as means ± standard deviations unless otherwise indicated. Participants who did not consume alcohol were classified as abstainers and those who consumed alcohol were classified as drinkers. Student *t*-tests and *chi*-square tests were used to determine differences between groups.

*HDL* high density lipoprotein, *AST* aspartate transaminase, *ALT* alanine aminotransferase, *IGI*_*60*_ 60-min insulinogenic index.

### Lipid profile and blood glucose index in association with *KCNJ11* rs5219 genotype according to alcohol consumption status

In drinkers, the IGI_60_ was marginally lower in the genotypes with at least one minor T allele than in the major homozygous C allele genotype (Table 1). Based on these findings, we performed genotype association analyses of *KCNJ11* rs5219 using the dominant model (major homozygous genotype [CC] vs. the risk allele group including the heterozygous [CT] and minor homozygous [TT] genotypes). Table 2 shows the lipid profiles and glycemic index values according to the *KCNJ11* rs5219 dominant model in association with alcohol consumption status. In the *KCNJ11* rs5219 major homozygous genotype, the lipid profile (total cholesterol, HDL-cholesterol, and triglycerides) and fasting glucose levels of the drinkers were higher than those of abstainers (*p* < 0.05), whereas insulin levels and IGI_60_ values were not different between the groups. Similarly, the total cholesterol, HDL-cholesterol, triglycerides, and fasting glucose levels of the drinkers were higher than those of abstainers in the risk allele group (*p* < 0.05); however, unlike the major homozygous genotype, the insulin levels and IGI_60_ were lower in the drinkers (6.8 mg/dL and 8.4 ± 8.0, respectively) than in the abstainers (7.2 mg/dL and 9.3 ± 8.5, respectively; *p* < 0.05).

**Table 2:** Lipid profiles and glycemic index according to *KCNJ11* genotype and alcohol consumption.

	CC		*p*-value	CT + TT		*p*-value
	Abstainers	Drinkers		Abstainers	Drinkers	
**Lipid profile**						
Total cholesterol (mg/dL)	187.9 ± 35.7	192.8 ± 36.1	0.0333	190.5 ± 34.6	194.1 ± 36.5	0.0434
HDL-cholesterol (mg/dL)	40.3 ± 8.3	44.9 ± 10.0	< 0.0001	40.2 ± 8.9	44.9 ± 10.2	< 0.0001
Triglycerides (mg/dL)	154.1 ± 83.0	180.2 ± 112.8	< 0.0001	163.8 ± 98.7	186.8 ± 132.6	< 0.0001
**Glycemic index**						
Glucose (mg/dL)	85.3 ± 14.4	89.8 ± 20.8	< 0.0001	85.9 ± 15.2	89.2 ± 18.2	< 0.0001
Insulin (mg/dL)	6.8 ± 3.4	6.7 ± 3.5	0.7559	7.2 ± 4.1	6.8 ± 3.3	0.0476
IGI_60_	9.3 ± 9.3	8.9 ± 8.8	0.5209	9.3 ± 8.5	8.4 ± 8.0	0.0472

Data are expressed as means ± standard deviations. Student *t*-tests were used to test for differences in lipid profile and glycemic index values among groups.

*HDL* high density lipoprotein, *IGI*_*60*_ 60-min insulinogenic index.

### Effects of *KCNJ11* rs5219 genotype on the risk for β-cell dysfunction depending on alcohol consumption status

Multivariate logistic regression analyses were performed to assess the association between the *KCNJ11* dominant model and risk for β-cell dysfunction in the abstainers and drinkers (Table 3). The risk for β-cell dysfunction was increased in the risk allele group (odds ratio [OR] 1.422; 95% confidence interval [CI] 1.100–1.839) compared to the major homozygous genotype. Furthermore, drinkers with risk alleles showed the increased risk for β-cell dysfunction compared to the major homozygous genotype (OR 1.450; 95% CI 1.061–1.982), whereas we found no association between β-cell function and the *KCNJ11* dominant model in the abstainers (OR 1.371; 95% CI 0.845–2.225).

**Table 3:** Effects of *KCNJ11* genotype and alcohol consumption on β-cell function.

	CC	CT + TT
All	Ref.	1.422 (1.100–1.839)
Abstainers	Ref.	1.371 (0.845–2.225)
Drinkers	Ref.	1.450 (1.061–1.982)

Multivariate logistic regression models were adjusted for age, smoking, and body mass index. Data are expressed as odds ratios (95% confidence intervals).

### Association between *KCNJ11* rs5219 genotype and insulin secretion according to long-term alcohol consumption

The baseline characteristics of participants according to the long-term alcohol consumption over the 12-year follow-up period are shown in Supplementary Table 3. Systolic and diastolic blood pressure, fasting glucose, HDL-cholesterol, triglycerides, and AST levels of drinkers were higher than those of never-drinkers (*p* < 0.05), whereas age, BMI, total-cholesterol, and ALT levels were not significantly different between the groups. The prevalence of T2D in drinkers was higher than that in never-drinkers (16.3% vs. 12.6%, respectively), although statistical significance was not observed. Finally, we investigated changes in insulin secretion in the *KCNJ11* rs5219 genotypes according to the long-term alcohol consumption. The mean IGI_60_ value was higher in never-drinkers (14.2) than in drinkers (10.3) with the major homozygous *KCNJ11* genotype and lower in drinkers than in never-drinkers with the risk alleles (9.9 vs. 11.4, respectively; Table 4).

**Table 4:** Comparison of insulinogenic index (60 min) values according to *KCNJ11* rs5219 genotype and the long-term alcohol consumption pattern.

	Never-drinkers	Drinkers	*p*-value
*KCNJ11* (rs5219, CC)	14.2 ± 12.8^a^	10.3 ± 8.6^a,b^	0.0374
*KCNJ11* (rs5219, CT + TT)	11.4 ± 10.0^a,b^	9.9 ± 8.8^b^	

Data are expressed as means ± standard deviations. Participants were classified as never drinkers or drinkers based on follow-up data collected over a 12-year period. General linear models were used to assess the relationship between *KCNJ11* genotype and alcohol consumption at follow-up on β-cell function (12-years).

^a, b^Significant differences among groups using the Duncan test.

## Discussions

Several meta-analyses and genome-wide association studies have reported an association between *KCNJ11* variant and the risk for T2D incidence or prevalence^10–12,14^. However, any studies did not show the effects of *KCNJ11* gene variant in relation with alcohol consumption status on the risk for T2D, especially on insulin secretion through long-term follow-up study. In this study, we for the first time investigated the association of *KCNJ11* rs5219 variant with alcohol consumption, and also with insulin secretion depending on alcohol consumption status, using baseline and 12-year follow-up data from a community-based Korean cohort.

It is clinically well known that blood glucose concentrations, insulin secretion, and insulin sensitivity change 3 to 6 years before the diagnosis of diabetes^15^. Decreased insulin secretion at the end stage of compensatory mechanism is mainly caused by dysfunction of β-cells, as one of major mechanism of T2D development^16^, increasing the risk for T2D incidence^17^. In this study, we estimated β-cell function by measuring the changes in plasma levels of glucose and insulin using the IGI_60_, which is commonly used to assess β-cell function. We found that the *KCNJ11* rs5219 risk allele group is significantly associated with reduced IGI_60_ values in response to alcohol consumption at both baseline and 12-year follow-up, but did not show statistical significance with insulin sensitivity and T2D. These findings may be due to the complexity of T2D, differences in genetic background and the frequency of risk alleles between different ethnic groups, and the other environmental factors. A previous meta-analysis reported that the presence of the rs5219 risk allele is significantly associated with increased T2D risk in East Asian and Caucasian populations, but not in Indian or other ethnic populations. Furthermore, the *KCNJ11* rs5219 variant is associated with the conversion from impaired glucose tolerance state to T2D development in Caucasians^14^ and reduces insulin secretion by overactivation of K_ATP_ channel^18^. Besides, the association between alcohol consumption and T2D can be explained by a decrease in insulin secretion. A cross-sectional study reported that insulin secretion is not significantly associated with alcohol consumption^19^, but other studies showed that alcohol consumption is associated with β-cell dysfunction^20,21^. Recently, our previous study has shown that through 12 years’ cohort study, insulin secretion was decreased, showing an inverted J-shape pattern depending on alcohol consumption pattern; meanwhile, the incidence risk of T2D showed J-shaped pattern^6^. Most previous studies suggested that light to moderate alcohol consumption are associated with a lower risk of diabetes mellitus^3,22^. The effects of alcohol consumption on β-cell function are still controversial. Nevertheless, alcohol consumption is obviously an important consideration part in the development of T2D.

The pancreatic β-cell K_ATP_ channel comprises two subunits, Kir6.2 and SUR1, which regulate glucose-stimulated insulin secretion. Both subunits are essential for K_ATP_ channel activity^23,24^. In the present study, we found neither an association between *KCNJ11* and *ABCC8* (gene that encodes the SUR1 protein) on insulin secretion, nor between *ABCC8* and insulin secretion according to alcohol consumption in Korean populations (Supplementary Tables 4 and 5). However, we observed changes in insulin secretion in the presence of the *KCNJ11* rs5219 variant according to alcohol consumption status in Korean men. There is a possibility that Kir6.2 is involved in the regulation of insulin secretion in response to alcohol consumption. To support this, SUR1-independent surface expression of a variant form of Kir6.2 (referred to as Kir6.2ΔC) did not affect ATP inhibition, meanwhile the expression of Kir6.2 alone was sensitive to ATP inhibition^25,26^. Moreover, a study that used site-directed mutagenesis identified a number of Kir6.2 residues that reduced ATP sensitivity of the K_ATP_ channel^27^. These findings suggest that the Kir6.2 subunit could be more critical to ATP sensitivity than the SUR1 subunit in regulation of insulin secretion by activating the K_ATP_ channel in β-cells. In addition, endosulfine alpha (ENSA) is an endogenous ligand for SUR1, which stimulates insulin secretion, and interacts with Kir6.2^28^. In term of impaired insulin secretion, the *KCNJ11* rs5219 is thought to affect the interruption of ENSA binding to SUR1 by altered Kir6.2 interaction. Further studies are needed to clarify the effects of the interaction between other genes including *KCNJ11* and alcohol consumption on insulin secretion.

Our study has some limitations. First, women were excluded from the study because little alcohol consumption data were available, as most were classified as abstainers; thus, we could not explore sex differences. Second, there may be self-report bias for alcohol consumption. Although a reliable and valid assessment of alcohol consumption requires objective measurement of alcohol intake using direct observation or breath alcohol analyses, those kinds of measures were not feasible in our study. Alternatively, we used blood indicators, including TG, AST, ALT and HDL-cholesterol to indirectly asses the alcohol consumption classifications. We hope our findings stimulate further research on the relationships among the *KCNJ11* rs5219 variant, alcohol consumption, and insulin secretion in other ethnic populations.

In summary, we found a significant association between *KCNJ11* rs5219 variant and decreased insulin secretion in alcohol drinkers at baseline and 12-year follow-up in a community-based Korean cohort. Our findings suggest that alcohol consumption in the presence of risk alleles decreases glucose-induced insulin secretion probably via impaired K_ATP_ channel activity, increasing the risk of T2D development. We hope that this study could be helpful to further understand the detail mechanism of T2D development through the interaction between genetic and environmental factors including alcohol consumption.

## Materials and methods

### Study population

Data were obtained from the community-based Ansan-Ansung Cohort Study conducted by the Korea National Institute of Health as part of the Korean Genome and Epidemiology Study (KoGES). In total, 4,182 men aged 40–69 years were recruited between 2001 and 2002 and followed-up by survey every 2 years^29^. Our study included data collected between 2001 and 2014. Women were excluded from the study because most abstained from alcohol use. In baseline data, individuals with missing data regarding β-cell function (insulinogenic index) by baseline (*n* = 1062) were excluded. In 12-years follow-up data, baseline was down β-cell function (*n* = 1557), missing data follow-up β-cell function (*n* = 756) were excluded. Concerning about long-term alcohol consumption, we excluded individuals who met at least one of the following criteria (*n* = 374); (i) those who responded to less than 80% of follow-up questionnaires about alcohol consumption, (ii) those who showed changes in their alcohol consumption pattern during the follow-up (failure to maintain the pattern of the initial questionnaire in consecutive examines), (iii) those who missed alcohol consumption information. The final analyses included baseline data of 3120 participants and 12-year follow-up data of 433 participants. Written informed consent was obtained from all participants. The study was approved by the National Biobank of Korea, the Centers for Disease Control and Prevention, Republic of Korea, and the study protocol was approved by the Korean National Institute of Health Institutional Review Board (2019-03-01-PE-A) and performed in accordance with approved guidelines.

### Anthropometric and biochemical analyses

Participant ages were obtained from interview-based questionnaires. BMI was calculated as weight divided by height squared. Blood pressure measurements were taken, and blood samples were drawn for biochemical measurements including glucose, insulin, total cholesterol, triglycerides, HDL-cholesterol, AST, ALT, and DNA. Pancreatic β-cell function was estimated using the 75 g oral glucose tolerance test (OGTT). The IGI_60_ was calculated as (insulin 60-insulin 0)/(glucose 60/glucose 0)^6,20^. T2D was defined as fasting glucose levels > 126 mg/dL or a 2 h post-OGTT glucose level > 200 mg/dL. Furthermore, participants who reported current use of antidiabetic medication or injected insulin were considered to have T2D^6^.

### Genotyping

The Ansan-Ansung Cohort Study was genotyped using the Affymetrix Genome-Wide Human SNP Array 5.0 and genotypes had been verified through quantile and quantile analysis, principal component analysis (PCA) and multidimensional scaling (MDS) analysis^30^. They were similar to those of the Japanese and Chinese components of HapMap. Genotype imputation was performed using IMPUTE2 software, and the East Asian ancestry sample of the 1000 genome project was used as the reference panel^31^. All genotype data were approved and provided by the National Biobank of Korea, the Centers for Disease Control and Prevention, Republic of Korea (No. 2019–022).

### Alcohol consumption measurement

Alcohol consumption data were collected at baseline and the biennial follow-up examinations using interview-based questionnaires. Participants were asked whether they had consumed at least one alcoholic drink every month, and if they had, they were asked whether they were former-drinkers or current-drinkers. Participants who did not consume alcohol at baseline were classified as abstainers and those who consumed alcohol were classified as drinkers. To better represent long-term alcohol consumption and to minimize within-person variation, we assessed the pattern of alcohol consumption using total daily alcohol consumption from baseline through the 12-year follow-up period^6^. Participants who did not consume alcohol during the entire follow-up period were classified as never-drinkers, and those who consumed alcohol during the follow-up period were classified as drinkers. The baseline and follow-up data of former drinkers were omitted from the analyses.

### Statistical analysis

Statistical tests were performed using SAS software (ver. 9.4; SAS Institute Inc., Cary, NC, USA). Data are expressed as means ± standard deviations, numbers (%), or odds ratios (ORs) with 95% confidence intervals (CIs). Logarithmic transformation was applied to variables with non-Gaussian distribution. Student t-tests were used to compare clinical characteristics between the alcohol consumption groups. Lipid profile and glycemic index values were used to assess differences in alcohol consumption according to the *KCNJ11* dominant model (CC vs. CT + TT). The *chi*-square test was used to compare categorical variables (*KCNJ11*, alcohol consumption, and T2D). Multiple logistic regression analyses were performed to investigate associations between β-cell function and the *KCNJ11* dominant model according to alcohol consumption after adjusting for age, smoking, and BMI. Analysis of variance (ANOVA, with Duncan’s post hoc test) was conducted to compare β-cell function according to long-term alcohol consumption and the *KCNJ11* dominant model. Statistical tests were two-tailed, and p-values < 0.05 were considered to indicate statistical significance.

## Supplementary Information

Supplementary Tables.
