# Supplementary material for: Association between KCNJ11 rs5219 variant and alcohol consumption on the effect of insulin secretion in a community-based Korean cohort: a 12-year follow-up study
Source: Sci Rep. 2021 Feb 25;11:4729. doi: 10.1038/s41598-021-84179-9 (PMC7907140; doi:10.1038/s41598-021-84179-9)
Supplement: Supplementary file 1 — Supplementary Tables. [file 41598_2021_84179_MOESM1_ESM.docx]

**Supplementary Tables 1, 2, 3, 4, and 5**

for “Association between *KCNJ11* rs5219 variant and alcohol consumption on the effect of insulin secretion in a community-based Korean cohort: A 12-year follow-up study” in *scientific reports*

Ji Ho Yun^1†^, Min-Gyu Yoo ^1†^, Ji Young Park^1^, Hye-Ja Lee^1*^, Sang Ick Park^1,*^

^1^Division of Endocrine and Metabolic Diseases, Center for Biomedical Science, Korea National Institute of Health, Cheongwon-gun, Chungcheongbuk-do, 363-951, South Korea

^†^**These authors contributed equally as co-first authors**

^*^**Correspondence to:**

Hye-Ja Lee, PhD

Phone: +82-43-719-8692, Fax: +82-43-719-8602, Email: [hyejalee@yahoo.co.kr](mailto:hyejalee@yahoo.co.kr)

Sang Ick Park, PhD

Phone: +82-43-719-8690, Fax: +82-43-719-8602, Email: [parksi61@hotmail.com](mailto:parksi61@hotmail.com)

Supplementary Table 1. Single nucleotide polymorphisms in *KCNJ11* gene and their frequencies

| Rs number | Position | Minor allele | Minor allele frequency |
| --- | --- | --- | --- |
| rs5210 | 17408251 | A | 0.4934 |
| rs5213 | 17408404 | C | 0.3955 |
| rs5215 | 17408630 | C | 0.3955 |
| rs5218 | 17409069 | A | 0.4329 |
| rs5219 | 17409572 | T | 0.3955 |
| rs5222 | 17410283 | T | 0.495 |
| rs35271178 | 17411020 | C | 0.3917 |
| rs886288 | 17411163 | A | 0.4896 |
| rs2074314 | 17411821 | C | 0.3894 |
| rs2074313 | 17412175 | G | 0.4932 |
| rs11024273 | 17412717 | T | 0.4932 |
| rs77023203 | 17412764 | G | 0.4901 |
| rs75780827 | 17412765 | C | 0.4928 |
| rs35762444 | 17412769 | C | 0.4868 |

Supplementary Table 2. Characteristics of the study participants according to *KCNJ11* genotype

|  | *KCNJ11* (rs5219) | | | *p*-value |
| --- | --- | --- | --- | --- |
|  | CC | CT | TT |  |
| Number of subject(%) | 1,128(36.2) | 1,514(48.5) | 478(15.3) |  |
| Age | 52.3±9.0 | 52.5±9.0 | 52.1±8.8 | 0.6620 |
| Body mass index | 24.6±3.1 | 24.5±3.1 | 24.6±2.9 | 0.5653 |
| Systolic blood pressure(mmHg) | 121.7±17.3 | 122.3±16.8 | 122.4±17.4 | 0.5860 |
| Diastolic blood pressure(mmHg) | 81.7±11.0 | 82.2±11.0 | 82.2±10.9 | 0.4709 |
| Glucose(mg/dL) | 88.4±19.1 | 88.3±17.4 | 88.2±17.9 | 0.9783 |
| HDL-cholesterol(mg/dL) | 43.5±9.7 | 43.7±10.2 | 43.4±9.7 | 0.8495 |
| Triglycerides(mg/dL) | 172.0±105.1 | 183.9±131.6 | 168.8±97.3 | 0.0084 |
| Total cholesterol(mg/dL) | 191.3±36.1 | 193.9±36.2 | 190.6±35.2 | 0.0895 |
| AST(IU/L) | 32.9±23.1 | 32.8±18.7 | 31.3±15.1 | 0.3079 |
| ALT(IU/L) | 33.2±27.6 | 34.0±27.8 | 31.5±18.8 | 0.1768 |
| IGI_60_ | 9.0±9.0 | 8.7±9.0 | 8.7±9.1 | 0.6063 |
| Type 2 Diabetes (%) | 93(8.2) | 125(8.3) | 38(8.0) | 0.7401 |
| Alcohol consumption (Drinker, %) | 775(68.7) | 1,100(72.7) | 330(69.0) | 0.0610 |

All data except type 2 diabetes and alcohol consumption are represented as mean ± standard deviation (SDs). Alcohol consumption levels were categorized into two group: participants who did not consume alcohol (Abstainers) and consume alcohol (Drinker). *P*-value were determined using Student *t*-test and chi-square test for baseline Characteristics in according to *KCNJ11*.

Supplementary Table 3. Baseline characteristics of the study participants by alcohol consumption pattern for 12-years follow-up

|  | Never-drinker | Drinker | *p*-value |
| --- | --- | --- | --- |
| Number of subject(%) | 114(19.4) | 474(80.6) |  |
| Age | 53.2±9.1 | 52.0±8.8 | 0.2732 |
| Body mass index | 24.0±3.1 | 24.6±3.1 | 0.1095 |
| Systolic blood pressure(mmHg) | 117.7±15.3 | 121.9±16.6 | 0.0133 |
| Diastolic blood pressure(mmHg) | 79.3±10.5 | 83.2±11.6 | 0.0012 |
| Glucose(mg/dL) | 83.2±9.1 | 86.4±10.6 | 0.0012 |
| HDL-cholesterol(mg/dL) | 39.2±7.0 | 44.2±9.5 | <0.0001 |
| Triglycerides(mg/dL) | 158.3±76.8 | 182.8±119.6 | 0.0071 |
| Total cholesterol(mg/dL) | 191.8±28.4 | 196.5±36.5 | 0.1335 |
| AST(IU/L) | 27.8±8.5 | 31.6±13.8 | 0.0002 |
| ALT(IU/L) | 31.0±18.4 | 34.4±21.9 | 0.0925 |
| Type 2 Diabetes (%) | 14(12.6) | 67(16.3) | 0.5537 |

All data except type 2 diabetes are represented as mean ± standard deviation (SDs). The participants were categorized into two groups based on follow-up measurement in Ansan-Ansung cohort (over 12-years alcohol consumption pattern). *P*-value were determined using Student *t*-test and chi-square test for baseline characteristics in according to alcohol consumption.

Supplementary Table 4. Effects of *ABCC8* (SUL1) rs2188966 genotype and alcohol consumption on β-cell function.

|  | CC | CG+GG |
| --- | --- | --- |
| All | Ref | 0.864(0.669-1.115) |
| Abstainers | Ref | 0.788(0.489-1.270) |
| Drinker | Ref | 0.894(0.660-1.210) |

Multivariate logistic regression models were adjusted for age, smoking, and body mass index. Data are expressed as odds ratios (95% confidence intervals).

Supplementary Table 5. Association between *KCNJ11* genotype and *ABCC8* (SUL1) genotype on β-cell function

|  | *KCNJ11* (rs5219) | | |  |
| --- | --- | --- | --- | --- |
|  | CC | CT | TT | *p*-value |
| ABCC8 (rs2188966) |  |  |  |  |
| CC | 8.5±8.2 | 8.4±8.8 | 8.7±8.9 | 0.9041 |
| CG | 9.6±9.7 | 8.7±8.7 | 8.9±9.6 | 0.2246 |
| GG | 8.8±8.7 | 9.8±10.4 | 7.8±7.2 | 0.3471 |
| *p*-value | 0.1267 | 0.1654 | 0.7281 |  |

All data are represented as mean ± standard deviations (SDs). *P*-value were determined using one-way ANOVA for association between *KCNJ11* and *ABCC8*.
